# Lower-dose intravenous immunoglobulin therapy for geriatric inflammatory bowel disease accompanied by COVID-19 multisystem inflammatory syndrome: A case report

**DOI:** 10.1097/MD.0000000000037888

**Published:** 2024-04-26

**Authors:** Peng Zhang, Jie Chen, Wenbin Zhao, Juan Liu

**Affiliations:** a Department of Neurology, PLA Joint Logistics Support Force No. 988 Hospital, Zhengzhou, China.

**Keywords:** case report, geriatric, inflammatory bowel disease, intravenous immunoglobulin, multisystem inflammatory syndrome, SARS-CoV-2 infection

## Abstract

**Rationale:**

This article presents a complex case of refractory severe acute respiratory syndrome coronavirus 2 (SARS-CoV-2)-related inflammatory bowel disease (IBD) and outlines its diagnostic and therapeutic challenges. Considering inadequate responses to conventional and steroid treatments, the potential efficacy of intravenous immunoglobulin is explored.

**Patient concerns:**

The patient, an elderly individual, experienced short-term fever and sore throat after encountering the coronavirus disease 2019 pandemic. Despite receiving a 3-dose inactivated SARS-CoV-2 vaccine, the patient tested positive for the viral antigen and developed worsening symptoms, including diarrhea and recurrent fever. Initial antibiotic treatment for bacterial enteritis proved ineffective.

**Diagnoses:**

Further evaluation, including endoscopy and pathology, confirmed the diagnosis of IBD with concurrent multisystem inflammatory syndrome (MIS) in adults, as evidenced by tachycardia and elevated inflammatory markers.

**Interventions:**

Following unsuccessful treatment with mesalazine, probiotics, corticosteroids, and supportive care, the patient underwent lower-dose intravenous immunoglobulin therapy.

**Outcomes:**

The patient experienced symptom improvement, with resolution of fever, diarrhea, and inflammation. At the 30-day follow-up, the patient remained afebrile, without diarrhea, and exhibited favorable mental status.

**Lessons:**

Elderly individuals infected with SARS-CoV-2 may develop severe systemic inflammatory responses. The patients in this report predominantly presented with IBD following SARS-CoV-2 infection, accompanied by MIS. Favorable clinical outcomes were achieved following lower-dose intravenous immunoglobulin immunotherapy, which demonstrated superior efficacy compared to glucocorticoids in managing such conditions. Future research should prioritize investigating immunotherapy application strategies in IBD and MIS. Notably, the significant clinical improvement observed with lower-dose intravenous immunoglobulin administration could optimize the utilization of this limited medical resource.

## 1. Introduction

The pervasive impact of coronavirus disease 2019 (COVID-19) is evidenced by its association with a postinfectious dysregulation of immune homeostasis, which simultaneously provokes the onset of inflammatory lesions. Though there have been instances of pediatric patients developing inflammatory bowel disease (IBD) as a sequela to the severe acute respiratory syndrome coronavirus 2 (SARS-CoV-2) infection,^[1]^ such occurrences in elderly patients remain sparingly reported. This article presents a noteworthy case of SARS-CoV-2-associated IBD in an elderly individual who endured 3 hospitalizations without favorable response to conventional treatment. Ultimately, the patient’s symptoms subsided following the administration of intravenous immunoglobulin (IVIg).

## 2. Case presentation

The study adhered to the principles outlined in the Declaration of Helsinki. Given the involvement of data from a vulnerable population, written informed consent was obtained from the patient himself, as well as his daughter, for the publication of this case report, which includes all data and images.

### 2.1. Case profile

An 80-year-old retired male presented at the hospital with a chief complaint of persistent diarrhea and fever spanning a period of 40 days. His medical history revealed the presence of a duodenal ulcer that had been successfully managed through standard treatment for a duration of 7 years. In addition, he had a medical history of coronary atherosclerotic heart disease and hypertension. He received percutaneous coronary stenting 3 years prior and maintained his cardiovascular health through daily doses of aspirin (100 mg), metoprolol tartrate (50 mg), atorvastatin calcium (10 mg), and amlodipine besylate (5 mg).

**Figure 1.**
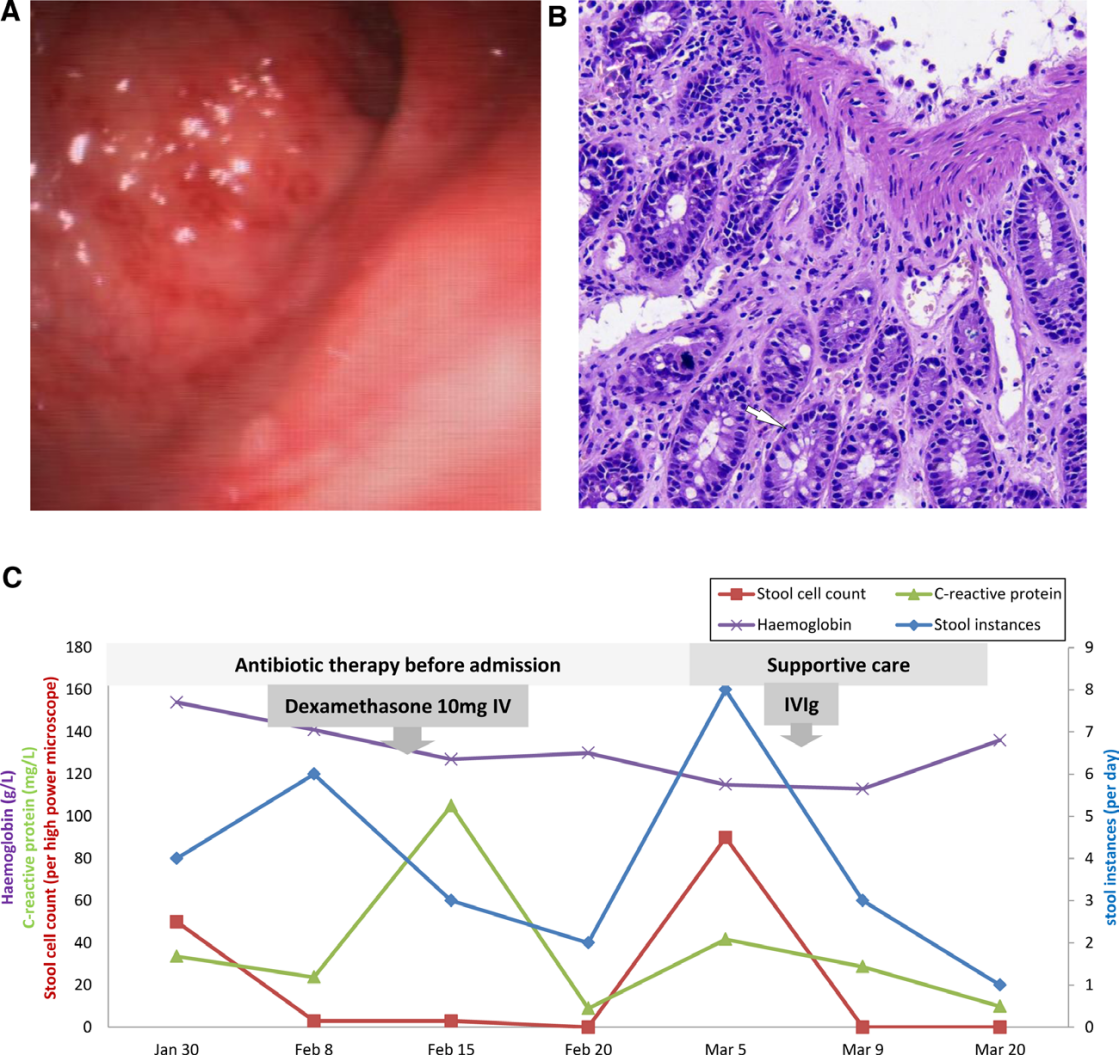
(A) Severe colitis (left) noted on endoscopy. (B) Chronic mucosal inflammation with multinucleated cell infiltration (right, arrow) (original magnification ×400). (C) Course of laboratory inflammatory markers (purple: hemoglobin, green: C-reactive protein, red: stool cell count, blue: stool instances, and treatment [gray bars and arrows]). IV, intravenous. IVIg, intravenous immunoglobulin.

The individual had a complex diagnostic and treatment trajectory prior to this admission. Despite receiving 3 doses of the inactivated Sinovac Biotech COVID-19 vaccine, he presented with transient fever and sore throat following exposure to a COVID-19 outbreak in early January 2023. Nasopharyngeal swab for SARS-CoV-2 antigen yielded a positive result. Subsequent changes in bowel habits from constipation to diarrhea, along with the recurrence of fever, were noted. By January 30, 2023, he demonstrated signs of possible progression to frank dysentery, characterized by blood and mucus in the stool. Laboratory examination revealed fecal red and white blood cell (WBC) counts of 50/HP and 80/HP, respectively, with a positive fecal occult blood test (FOBT). His total WBC count remained within normal limits; however, there were observed increases in neutrophil percentage, C-reactive protein (CRP), and procalcitonin. Given clinical manifestations congruent with bacterial enteritis, initial treatment with levofloxacin (0.2 g/d) was prescribed for 7 days in primary care. Despite this, clinical symptoms persisted, and FOBT remained positive, necessitating his first hospital admission. A working diagnosis favored bacterial enteritis over ulcerative colitis (UC), and antibiotic therapy was reinitiated. Subsequent supportive care provided temporal stabilization, permitting further gastrointestinal evaluation. Esophagogastroduodenoscopy identified chronic atrophic gastritis, while colonoscopy revealed erosive colitis and rectitis. Biopsies from the transverse colon showed chronic mucosal inflammations (Fig. 1). Unexpectedly, the patient’s fever returned, with CRP surging to 105 mg/L. Following treatment with intravenous dexamethasone (10 mg/d for 5 days) and another course of antibiotics (cefoperazone sodium sulbactam sodium, Pfizer, USA), primary symptoms abated. The patient was discharged on a regimen of mesalazine and probiotics, only to return 10 days later with severe diarrhea (up to 8 instances per day) and intermittent febrile episodes reaching 39°C, which necessitated his readmission. His condition failed to improve over the ensuing days, prompting a transfer to our facility. Given newly developed symptoms of dizziness and fatigue, combined with his existing vascular risk factors, he was admitted to our neurology department.

### 2.2. Physical examination and auxiliary examination

Clinical examination revealed the patient to be underweight, lethargic, and dehydrated, with no significant neurological deficits. Vital signs were notable for fever (maximum temperature of 38.1°C), hypotension (95/56 mm Hg), tachycardia (115 beats/min), an elevated respiratory rate (22 breaths/min), and an oxygen saturation of 97% on room air. Further assessment showed edema in both lower extremities. He exhibited no abdominal tenderness upon palpation, hepatosplenomegaly, respiratory distress, lymphadenopathy, jaundice, conjunctivitis, joint tenderness or swelling, and had a normal perianal examination.

Laboratory analysis revealed significant hypoproteinemia, with total protein and albumin levels recorded at 49.3 g/L (reference range: 60–82 g/L) and 27.8 g/L (reference range: 35–54 g/L), respectively. Mild anemia was detected, with a hemoglobin concentration of 115 g/L (reference range: 120–160 g/L) and a mean corpuscular volume of 98.9 fL (reference range: 80–100 fL). The patient exhibited electrolyte imbalances, displaying both hypokalemia and hyponatremia with levels of 3.0 mmol/L (reference range: 3.5–5.3 mmol/L) and 135 mmol/L (reference range: 135–145 mmol/L), respectively. The patient’s inflammation markers suggested an ongoing inflammatory response, with a WBC count of 6610/μL (reference range: 6000–10,000/μL), neutrophil percentage of 84.6% (reference range: 50–70%), platelet count of 228,000/μL (reference range: 100,000–300,000/μL), erythrocyte sedimentation rate of 23 mm/H (reference range: 0–15 mm/H), CRP level of 41.7 mg/L (reference range: 0–10 mg/L), procalcitonin level of 0.54 ng/mL (reference range: ≤0.5 ng/mL), and an interleukin-6 level of 28.77 pg/mL (reference range: 0–7 pg/mL). His international normalized ratio was 1.12 (reference range: 0.8–1.2) and the D-dimer level was elevated at 677 ng/mL (reference range: ≤200 ng/mL). Stool microscopy revealed a fecal white blood cell count of 90/HP, a fecal red blood cell count of 3/HP, and a positive FOBT. However, tests for infectious agents, including stool and blood cultures, *Clostridium difficile* testing, and nasopharyngeal swab for SARS-CoV-2 polymerase chain reaction (PCR), returned negative results. Additional diagnostic tests, encompassing tuberculosis antibody testing, Brucella testing, and autoantibody lineage testing were also negative. The immunoglobulin and complement assays yielded results within normal parameters. Comprehensive neuroimaging, pulmonary evaluation, and ultrasound assessments of the heart and abdomen were unremarkable. The electrocardiogram displayed sinus tachycardia. A proposal to repeat the digestive endoscopy was not approved.

### 2.3. Diagnostic process and treatment approaches

The patient met the clinical criteria for IBD with concurrent multisystem inflammatory syndrome (MIS) in adults.^[2]^ On the third day of hospitalization, his hypotensive state and electrolyte abnormalities were rectified through the administration of normal saline boluses and nutritional support. However, persistent fever, diarrhea, and tachycardia remained unresolved. Given the lack of enduring efficacy with prior corticosteroid treatment and potential adverse effects for this elderly patient with underlying comorbidities, IVIg therapy was initiated at 0.2 g/kg. Adjunctive interventions comprised intravenous human serum albumin and oral administration of mesalazine and probiotics. After 2 days of IVIg therapy, the patient demonstrated notable improvement in fever and diarrhea symptoms.

### 2.4. Prognosis and follow-up

Although the IVIg treatment was discontinued after 4 days due to resource constraints, the patient’s condition continued to enhance. Fever and diarrhea ceased, while dizziness and lower extremity edema resolved. Fatigue markedly reduced, permitting the patient to ambulate. Stool microscopic analysis indicated the absence of detectable white and red cells. Within 10 days, his serum inflammatory markers returned to normal values. Neutrophils reduced from 84.6% to 55.8% with a WBC count of 7170/μL, and the CRP level decreased from 41.7 to 9.9 mg/L. Hemoglobin levels increased to 135 g/L. Erythrocyte sedimentation rate, D-dimer, total protein, and albumin levels also returned to their respective normal ranges. He was discharged on the 16th day of hospitalization and has continued to demonstrate improvement post-discharge. At the 30-day follow-up, the patient remained afebrile, devoid of diarrhea, and exhibited a favorable mental status. Mesalazine and probiotics were discontinued. His stool characteristics returned to the pre-COVID-19 infection state, and his weight increased to 65 kg.

## 3. Discussion

Persistent diarrhea, bloody stools, fever, and abnormal inflammatory markers indicated intestinal inflammation. However, the ineffectiveness of antibiotic therapy in managing his diarrhea and the absence of pathogenic bacteria in repeated stool cultures made bacterial enteritis an unlikely diagnosis. Further insights were obtained from colonoscopy findings, which revealed a patchy appearance across the entire colon, mucosal edema, and erosions. Histological analysis of the colonic mucosa aligned with these findings, leading to a diagnosis that fulfilled the clinical criteria for IBD,^[3]^ with a predilection towards UC. In contrast to Crohn disease, the other primary subtype of IBD, UC typically presents a shorter disease course, lacks characteristic intestinal structural abnormalities, and responds more favorably to immunomodulatory therapies. The involvement of the rectum in UC might elucidate the observed increase in the frequency of bowel movements, attributable to rectal irritation, and a sensation of tenesmus.

The exact etiology of IBD remains elusive, though it is generally accepted that a convergence of genetic, environmental, and immunological factors contributes to its manifestation. First, individuals possessing a familial history of IBD are at an increased risk of developing the condition. In this case, the patient reported no such family history, and his prior duodenal ulcers were deemed successfully treated, as confirmed by endoscopic evaluations. Nonetheless, no additional genetic susceptibility screening was performed, including the assessment of notable IBD-related genes.^[4]^ Second, the intestinal microbiota stands as a significant environmental contributor to IBD. While repeated stool bacteriological analyses did not yield any discernible pathogenic organisms, it is crucial not to completely exclude the possibility of a prodromal bacterial infection. IBD is unlikely to be caused or triggered by a singular infection in humans,^[5]^ but the intestinal microbiota clearly plays a significant role in its development. The patient’s stool microscopy did not reveal a dysbiosis of coccidiobacteria, yet without a comprehensive gut microbiome assessment, potential microbiota imbalances secondary to multiple rounds of antibiotic treatment cannot be completely ruled out. Considering this possibility, probiotics were administered until discharge. Additionally, the timing of nonsteroidal anti-inflammatory drugs administration could exacerbate intestinal injury and perturb the microbiome.^[6]^ To circumvent potential induction of IBD activity, lower-dose aspirin therapy—part of the patient’s coronary artery disease management—was discontinued until diarrhea symptoms stabilized. Patel’s retrospective analysis, however, suggests that daily lower-dose aspirin, distinct from general nonsteroidal anti-inflammatory drugs usage, does not significantly increase the likelihood of IBD-related hospitalization.^[7]^ Third, immunological anomalies, typically involving dysregulation of either innate or adaptive immunity, have been implicated in the pathogenesis of IBD. The patient presented multi-organ symptoms such as diarrhea, tachycardia, and dizziness following prodromal SARS-CoV-2 infection. In addition, persistent fever, elevated levels of CRP, interleukin-6, and procalcitonin led us to consider a potential association with COVID-19-related MIS in adults, where IBD was the predominant presentation.

The principal pathogenic mechanisms of MIS include immune dysregulation, cytokine storm, endothelial dysfunction, autoimmunity, and genetic predisposition. MIS is a rare yet severe condition associated with COVID-19, characterized by inflammation affecting various internal and external body parts, including the heart, lungs, kidneys, brain, skin, eyes, and gastrointestinal tract. It may be speculated that an aberrant immune response to a preceding viral infection, most commonly SARS-CoV-2, triggered the manifestation of IBD in this susceptible host. Subsequent cytokine storm—characterized by an excessive release of pro-inflammatory cytokines—could have induced widespread inflammation and damage to multiple organs. While MIS predominantly affects children and adolescents, this case underlines that elderly patients may also constitute a susceptible group for COVID-19-related MIS.

The diagnosis of MIS greatly influenced the choice of treatment, with IVIg chosen as the key intervention. The effectiveness of IVIg for the treatment of IBD remains under investigation and it is not currently considered a standard or first-line therapy for this condition. Nevertheless, IVIg has been observed to yield favorable clinical outcomes in cases of refractory IBD.^[8,9]^ Concurrently, the efficacy of IVIg in treating MIS in children is well established and it is regarded as a crucial component of the treatment strategy for MIS.^[10]^ IVIg can modulate the immune system by interacting with a variety of components including B cells, T cells, natural killer cells, and antigen-presenting cells. It can suppress excessive immune activation and attenuate inflammatory responses. IVIg also contains a high concentration of antibodies, including immunoglobulin G. These antibodies can bind to pathogens, toxins, or autoantibodies, neutralizing their effects and aiding their clearance from the body.^[11]^ In this case, the patient received a half-dose of IVIg, which led to a good prognosis, and alternative treatments like corticosteroids and infliximab were not initiated. This might suggest that the immune-related inflammation caused by COVID-19 is different from relapsing-remitting autoimmune diseases. Early application of even low doses of IVIg can exert a significant immunomodulatory effect and slow down the immune-related inflammatory response.

This study possesses certain potential limitations. First, despite conducting an extensive workup to exclude concurrent infectious etiology, PCR-based gastrointestinal pathogen testing could have been performed for a more comprehensive understanding. Regrettably, our request for a repeat colonoscopy was not authorized by the patient. Second, although the patient tested negative for SARS-CoV-2 virus via PCR test after admission, the prodromal SARS-CoV-2 viral infection was evident. The patient’s inflammatory manifestations were managed as systemic inflammatory response syndrome rather than COVID-19 MIS-A and did not impact the treatment decision in this instance.

## 4. Conclusions

The presence of a prodromal SARS-CoV-2 infection and the subsequent development of MIS further complicated the clinical presentation. The successful use of IVIg in this case suggests its potential efficacy in treating refractory IBD with concurrent COVID-19 MIS in elderly patients. Enhanced comprehension of the interplay between COVID-19 infection, MIS, and autoimmune diseases is imperative for refining diagnosis and treatment strategies in affected individuals.

## Author contributions

**Conceptualization:** Peng Zhang, Jie Chen.

**Data curation:** Wenbin Zhao, Juan Liu.

**Investigation:** Wenbin Zhao, Juan Liu.

**Writing – original draft:** Peng Zhang.

**Writing – review & editing:** Jie Chen.
